# Development of an Intelligent Clinical Decision Support System for Spirometry Quality Control

**DOI:** 10.3390/diagnostics16020213

**Published:** 2026-01-09

**Authors:** Julia López-Canay, Ana Priegue-Carrera, Alejandro Casado-Trigo, Cristina Represas-Represas, Alberto Fernández-García, Alberto Comesaña-Campos, Manuel Casal-Guisande, Alberto Fernández-Villar

**Affiliations:** 1 NeumoVigo I+i Research Group, Galicia Sur Health Research Institute (IIS Galicia Sur), SERGAS-UVIGO, 36312 Vigo, Spain; julia.lopez@iisgaliciasur.es (J.L.-C.); ana.priegue@iisgaliciasur.es (A.P.-C.); acasado246@gmail.com (A.C.-T.); cristina.represas.represas@sergas.es (C.R.-R.); acomesana@uvigo.es (A.C.-C.); jose.alberto.fernandez.villar@sergas.es (A.F.-V.); 2 Pulmonary Department, Hospital Álvaro Cunqueiro, 36312 Vigo, Spain; 3 Centro de Investigación Biomédica en Red, CIBERES ISCIII, 28029 Madrid, Spain; 4 Diagnostic Imaging Department, Hospital Ribera Povisa, 36211 Vigo, Spain; alberto.fernandez.garcia@outlook.es; 5 Department of Design in Engineering, University of Vigo, 36208 Vigo, Spain; 6 School of Industrial Engineering, University of Vigo, 36310 Vigo, Spain

**Keywords:** spirometry, quality control, intelligent system, deep learning, convolutional neural networks, spirometry curves

## Abstract

**Background**: Spirometry is the most widely used pulmonary function test for diagnosing respiratory diseases. However, the quality of the results mainly depends on the correct execution of the maneuver, making quality control essential. Traditionally, this process relies on subjective and laborious visual inspection. **Methods**: To overcome these limitations, this work proposes an intelligent clinical decision support system to assist in spirometry quality control. The proposed system generates a graphical construct that integrates the spirometry curves (flow-volume and volume-time curves) along with patient demographic information (sex, age, and BMI) extracted from the spirometry report. The resulting image is processed by a convolutional neural network based on the ResNet-18 architecture, whose output quantifies the risk of the performed test being unacceptable. This approach allows for simple integration of the system into clinical practice while accounting for individual patient characteristics during classification. **Results**: The results obtained in the test set are promising, with an AUC of 0.94 (95% CI: 0.80–1.00) and a sensitivity and specificity at the selected cut-off point of 75.00% (95% CI: 40–100%) and 100.00% (95% CI: 100–100%), respectively. **Conclusions**: Despite this, it should be noted that the system is still in a conceptual phase of development and therefore requires broader validation in real clinical environments as well as the incorporation of more diverse datasets to evaluate its robustness and generalization before its final implementation.

## 1. Introduction

Spirometry is a physiological test that evaluates the maximum flow and volume of air a person can exhale during a maximal expiratory effort [1]. It is the most used pulmonary function test due to its fundamental role in the diagnosis, monitoring, and management of respiratory diseases such as asthma and chronic obstructive pulmonary disease (COPD) [1,2,3]. Currently, the growing prevalence of chronic pulmonary diseases [4], along with the increased accessibility to spirometers, has favored its expansion beyond specialized services, incorporating it into primary care and even non-clinical settings, such as pharmacies [5,6,7]. Its implementation at the primary care level allows for an early diagnosis and staging of COPD [8,9], as well as the confirmation of asthma [7].

Spirometry is a simple, non-invasive, safe, and low-cost test; however, its quality can be compromised by multiple personal and technical factors [10]. The correct execution of the test largely depends on the active cooperation of the patient and the proper training of the responsible technician [1]. Poor execution can generate false positives in the diagnosis of airway obstruction, which leads to an inappropriate diagnostic and therapeutic approach, with possible negative consequences for the patient, such as unnecessary stress or adverse effects derived from the treatment [7].

In this context, the American Thoracic Society (ATS) and the European Respiratory Society (ERS) [1,10,11] have established standards that define specific numerical parameters for spirometry quality control. Nevertheless, the visual inspection of the spirometry curves (flow-volume and volume-time) is still considered the gold standard for test evaluation [1]. This procedure, however, is subjective and time-consuming, which implies variability in the results [12]; in fact, the degree of agreement among professionals can be as low as 52% [13]. Furthermore, the increased availability of spirometers in primary care has not been accompanied by systematic training programs for professionals, which have been shown to be effective in improving the quality of testing [14,15]. In fact, quality standards for COPD care indicate that spirometry should be performed by trained personnel, and its validity must be ensured [16]. Despite this, studies such as that by Hueto et al. [2] show that only 64% of the professionals who perform spirometry in this setting have received specialized training.

In recent years, Artificial Intelligence (AI) has transformed the healthcare field, providing systems that seek to support clinical decision-making processes [17,18,19,20]. Recent literature indicates that these tools can be especially useful in assisting primary care professionals with the quality assessment of spirometry tests. Specifically, various proposals have been developed that use Deep Learning (DL) techniques involving convolutional neural networks (CNNs), which have demonstrated notable success in image classification tasks [21,22].

Walag et al. [23] used a CNN to process images of the flow-volume and volume-time curves, combining it with a rule-based module to determine the acceptability and usability of the following spirometry parameters: forced expiratory volume in the first second (FEV_1_) and forced vital capacity (FVC). The developed classifiers demonstrated promising results, with sensitivities ranging from 97.5% to 99.5% and specificities between 85.4% and 92.4%. On the other hand, Das et al. [12] employed a CNN to classify spirometry tests as acceptable or unacceptable using images of the flow-volume curve, achieving an area under the ROC curve (AUC) of 0.93. Additionally, they developed a specific classifier to assess the usability of the FEV_1_ parameter, achieving an AUC of 0.98. Finally, Bonthada et al. [24] used two independent CNNs to detect errors in spirometry tests and classify them by error type: extra breaths, high extrapolated volume, early termination, or submaximal effort. For the detection of error presence, they achieved a sensitivity of 98% and a specificity of 87%.

Despite the promising results, studies in this field are still limited and show high heterogeneity in methodology and classification objectives [25]. The developed systems are designed to determine the overall acceptability of the test, the acceptability of specific parameters, or the detection of errors, and they use different types of information, including spirometry parameters and images of the flow-volume and volume-time curves. However, this information is usually processed by independent modules, when its integration could improve the performance of the classifiers. Furthermore, no study has incorporated demographic parameters, which are routinely included in spirometry reports and could influence test performance. Finally, no system has been developed specifically for primary care, where its implementation is most relevant, which could limit their effectiveness if applied in this healthcare environment.

In this context, the present work proposes the design and development of an intelligent system for spirometry quality control, based on a CNN capable of classifying tests as acceptable or unacceptable. The system has been developed specifically for its use in primary care, using a database that contains spirometry reports obtained within the framework of training courses aimed at healthcare professionals working in non-specialized settings. With the aim of optimizing classifier performance, a graphical construct was defined that integrates demographic information included in the spirometry report—body mass index (BMI), sex, and age—along with the flow-volume and volume-time curves. The encoding and integration of tabular data into images for their processing through deep architectures constitutes an appropriate and increasingly adopted strategy in the state of the art, as it enables the integration of multidimensional information (see, for example, the work by Zhu et al. [26], where variables are encoded using sets of pixels with different values or intensities). Finally, this graphical construct is processed by the CNN to determine the acceptability of the test. The main contributions of this article are the following:

• To propose the architecture of a new intelligent system to assist in spirometry quality control, capable of classifying tests as acceptable or unacceptable based on the spirometry report.
• To develop a graphical construct that integrates the flow-volume and volume-time curves along with relevant patient demographic information reflected in the report, with the aim of serving as input for a CNN and classifying the tests.
• To implement the system in a software artifact and evaluate its practical applicability with a case study.

The article is organized into 5 sections. Section 1 defines the framework of the proposed system. In Section 2, Section 2.1 presents the database used for the system’s development. Subsequently, Section 2.2 and Section 2.3 detail the conceptual design and implementation of the intelligent system. Section 3 presents a case study that exemplifies the system’s operation as a proof of concept. Finally, Section 4 offers a discussion of the work, and Section 5 presents the conclusions.

## 2. Materials and Methods

### 2.1. Database Usage

A database consisting of 282 fully anonymized spirometry records was used for the construction of the intelligent system. These tests come from spirometry training courses aimed at healthcare professionals working in non-specialized settings within the Vigo Healthcare Area. The equipment used to perform the tests was a Medikro^®^ Pro M915 spirometer (Kuopio, Finland).

The obtained reports were sent to Álvaro Cunqueiro Hospital in Vigo, where quality control was performed according to the standards established by the ATS/ERS [1,10,11].

Each spirometry report consists of several pages. The first page, which is used for test evaluation, is divided into several sections: personal and clinical information, smoking habits, test results, flow-volume and volume-time curves and the interpretation of the test.

The reports were individually reviewed by experts in the field, with the aim of determining the acceptability of the tests. For this purpose, a visual inspection of the flow-volume and volume-time curves was performed, assigning label 0 to non-valid tests and label 1 to valid tests.

After the labeling and data cleansing phase, 224 records were selected and used for the system’s training and evaluation, of which 79 were unacceptable tests and 145 were acceptable tests, as summarized in Table 1.

To define the graphical construct, relevant information contained in the reports was selected considering the experience of healthcare professionals. Thus, the flow-volume and volume-time curves were used, along with patient demographic data, which include sex, age, and BMI. Table 2 shows a summary of the characteristics of these demographic variables.

### 2.2. Conceptual Design

**Figure 1:**
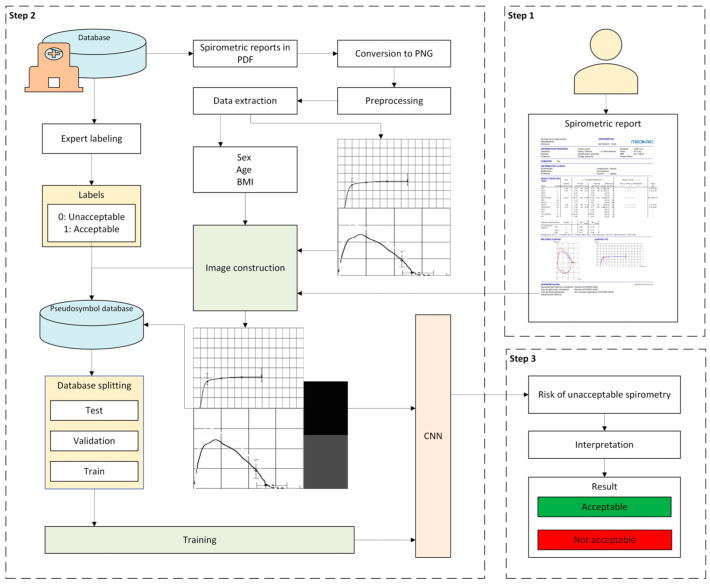
Flow diagram of the intelligent system.

Figure 1 shows the flow diagram of the intelligent system applied to spirometry quality control developed in this work. A detailed description of the defined stages is provided below.

#### 2.2.1. Data Collection

First, patient data collection is performed. For this, the report corresponding to the spirometry test performed by the patient must be entered into the system. As detailed in Section 2.1, the report must include the images of the flow-volume and volume-time curves, as well as the patient’s demographic information: sex, age, and BMI.

#### 2.2.2. Data Processing

Once the patient data has been collected, it is processed by the system. For this purpose, a graphical construct is defined. This construct integrates all the patient’s information into a single image. The construction process is developed in the following stages:

• First, the most relevant features contained in the spirometry report are selected according to expert criteria. These include demographic data (BMI, sex, and age), as well as the images of the flow-volume and volume-time curves. The images of the flow-volume and volume-time curves are filtered, converted to grayscale and combined into a single composite image.
• Subsequently, this image is enriched with the patient’s demographic information. The numerical variables (BMI and age) are normalized using the MIN-MAX method, adjusting their values to the range [0, 1]. The categorical variable (sex) is encoded by assigning the value “0” to man and “1” to woman. This information is integrated into the image by generating a unique graphical construct through the addition of a frame on the right side divided into three sections. Each section corresponds to one of the demographic variables, whose grayscale level represents the variable’s value.

Finally, the resulting image is used as input for a CNN, which acts as a binary classifier, obtaining at its output the risk that the performed test is unacceptable.

#### 2.2.3. Alert Generation and Decision Making

Once the data has been processed, the risk associated with a spirometry being unacceptable is obtained. Based on this information, the test is classified as acceptable or unacceptable, providing the personnel in charge with the necessary details for appropriate patient management.

### 2.3. Implementation of the System

The implementation of the system was carried out in the MATLAB© environment (R2024a version, Natick, MA, USA). The Deep Learning Toolbox [27] was used for the system’s development, specifically for the design, training, and implementation of the CNNs, as well as the Image Processing Toolbox [28], intended for the preprocessing and manipulation of spirometry data. Additionally, the Text Analytics Toolbox [29] was employed for automatic text extraction from the PDF reports, and the App Designer module [30] for developing the system’s interactive graphical interface. Furthermore, the Java Apache PDFBox library [31] was used for handling the reports in PDF format. The equipment used for training the CNN features an Apple M2 Pro processor (Cupertino, CA, USA), with an integrated 16-core GPU and 16 GB of RAM.

**Figure 2:**
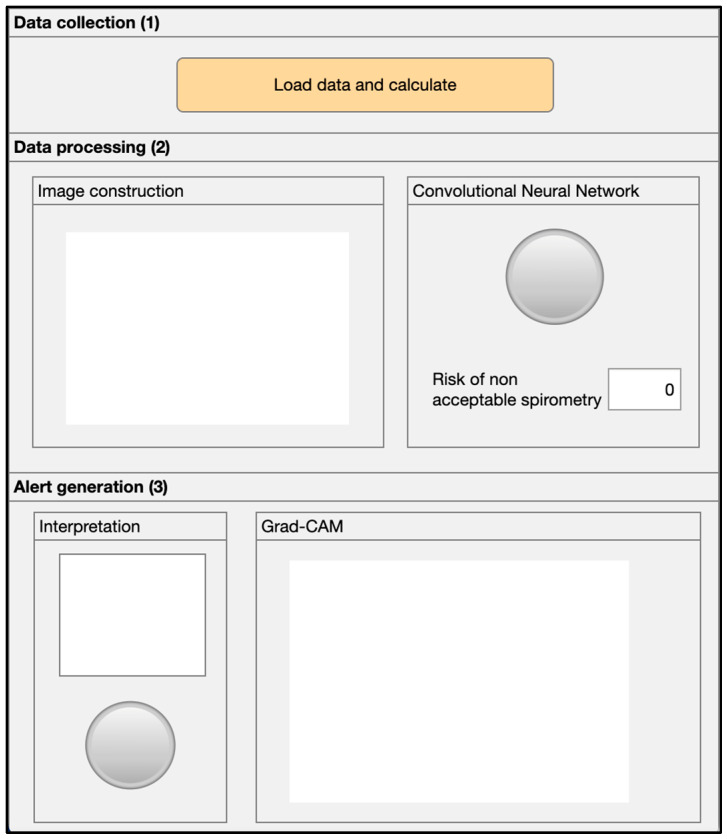
Screenshot of the intelligent system’s user interface.

Figure 2 shows a screenshot of the main program, where the following areas are distinguished: (1) data collection area, (2) data processing area, and (3) alert generation area.

#### 2.3.1. Data Collection

First, the spirometry report corresponding to the test performed by the patient is loaded into the application via section (1) of the user interface, as shown in Figure 2.

#### 2.3.2. Data Processing

Once the patient information is collected, it is processed by the intelligent system. First, the relevant data is extracted, including the spirometry curves and the patient’s demographic data (BMI, age, and sex). Next, this information is processed and integrated into a unique graphical construct, which serves as input for a CNN. The output of the network corresponds to the risk that the performed spirometry test is unacceptable.

##### Image Construction

**Figure 3:**
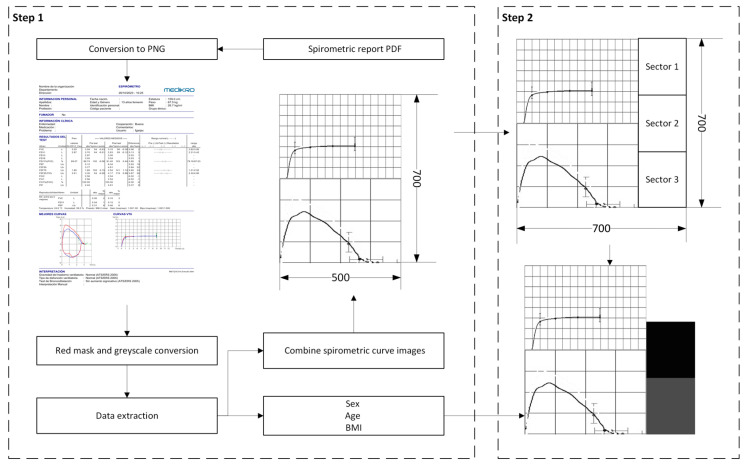
Image construction process.

The construction of the image is carried out in two stages, as shown in Figure 3. A detailed description of the process is provided below.

First, to facilitate the system’s implementation, a preprocessing of the variables was carried out. In this case, the numerical variables (BMI and age) were normalized using the MIN-MAX method, adjusting their values to the range [0, 1]. For the variable age, minimum and maximum values of 10 and 84 years were selected, while for the variable BMI, values of 15 and 54 kg/m^2^ were used. On the other hand, the sex variable was encoded by assigning the value “0” to man and “1” to woman.

• Step 1: in this step, the images of the flow-volume and volume-time curves are extracted from the spirometry report, which is in PDF format. First, only the first page of the report, where the curves of interest are located, is kept. Then, this page is converted to a PNG image for its preprocessing. The flow-volume and volume-time curves may appear in two forms: a single curve (blue color) or two superimposed curves (blue and red). The red curve corresponds to a spirometry performed after the administration of a bronchodilator and therefore must be removed. To do this, a color mask is applied to eliminate this curve by transforming the red, maroon, and pink tones into white. Following the conversion of the entire image to grayscale, the regions of interest containing the curves are extracted. For this purpose, the image is divided horizontally, and only the bottom half of the report, where the graphs are located, is retained. This section is binarized and inverted, so that the curves appear in white on a black background. Next, a filter is applied to remove noise, discarding small regions with an area less than 1000 pixels. This threshold allows for the suppression of small spots or artifacts without affecting the main curve. Additionally, a closing operation is performed to fill gaps and unify strokes in case there were overlaps between the target curve and the curve obtained after bronchodilator administration. This process ensures that no information is lost after applying the color mask. Finally, the connected regions in the binary image are detected, and their bounding boxes are obtained. The regions are ordered by area, keeping the two largest, which correspond to the flow-volume and volume-time curves. Each curve is individually cropped and stored in a new 700 × 500 pixel image, placing the volume-time curve at the top and the flow-volume curve at the bottom.
• Step 2: in this step, the image is enriched by incorporating the patient’s demographic information (sex, age, and BMI), to build the graphical construct that will serve as input to the system. To do this, an additional frame is added to the right side of the previously generated image, expanding its dimensions to obtain a square image of 700 × 700 pixels. This frame is divided into three equal sections, each corresponding to one of the demographic variables. Each section is encoded in grayscale according to the normalized value of the variable it represents. Specifically, the shade of gray is generated by assigning the Red (R), Green (G), and Blue (B) channels a value equal to the normalized variable value multiplied by 255. Finally, the image is resized to 224 × 224 pixels, which is the typical size for neural network architectures such as ResNet-18 [32].

##### Convolutional Neural Network

Once the image is obtained, it is processed by a CNN. For this purpose, the network must be trained beforehand.

The CNN adjustment process was carried out using a total of 224 images, of which 90% (202) were allocated for training and the remaining 10% (22) were reserved as a test set to evaluate the model’s generalization capability. Additionally, 23 images of the training set were reserved for network validation during the training process.

The dataset used presents certain limitations, notably its limited size and class imbalance. To mitigate the effects of both issues, a data augmentation strategy based on the application of random geometric transformations to the images during training was implemented. This technique increases the variability of the dataset and improves the generalization capacity of the model. Table 3 details the specific transformations applied during the data augmentation process.

Additionally, a transfer learning strategy was employed with the purpose of avoiding the random initialization of the weights. In this case, the ResNet-18 architecture [32], previously trained on a subset of the ImageNet database, was used. To adapt the network to the specific problem, the output layer was modified to allow for the construction of a binary classifier.

The CNN was trained using the Deep Learning Toolbox [27] from MATLAB. Table 4 shows the configuration employed for training.

Once the network’s training is complete, its output corresponds to the risk that a spirometry is unacceptable. The value of this risk is expressed in the range [0, 1].

The network functions as a binary classifier. To determine, based on the risk obtained as output, whether the test should be assigned the label “Acceptable Test” or “Unacceptable Test”, it is necessary to establish a threshold value for the classification. For this purpose, the Matthews Correlation Coefficient (MCC) method [33,34,35] was used, as employed by Casal-Guisande et al. in other studies [36]. This method allows a comprehensive evaluation of the model’s performance, assigning a high score only when satisfactory results are achieved in all categories of the confusion matrix [34]. The MCC ranges from [−1, 1], with 1 representing a perfect prediction, −1 an inverse prediction and 0 a prediction based on chance [34]. For this reason, it is an ideal metric for selecting an optimal cutoff point while working with an imbalanced database. Equation 1 contains the expression used for the calculation of the MCC, where TN = True Negatives, FN = False Negatives, TP = True Positives, and FP = False Positives.

(1) $$Mcc=\frac{TN\cdot TP-FN\cdot FP}{\sqrt{\left(TP+FP)\cdot (TP+FN)\cdot (TN+FP)\cdot (TN+FN\right)}}$$

**Figure 4:**
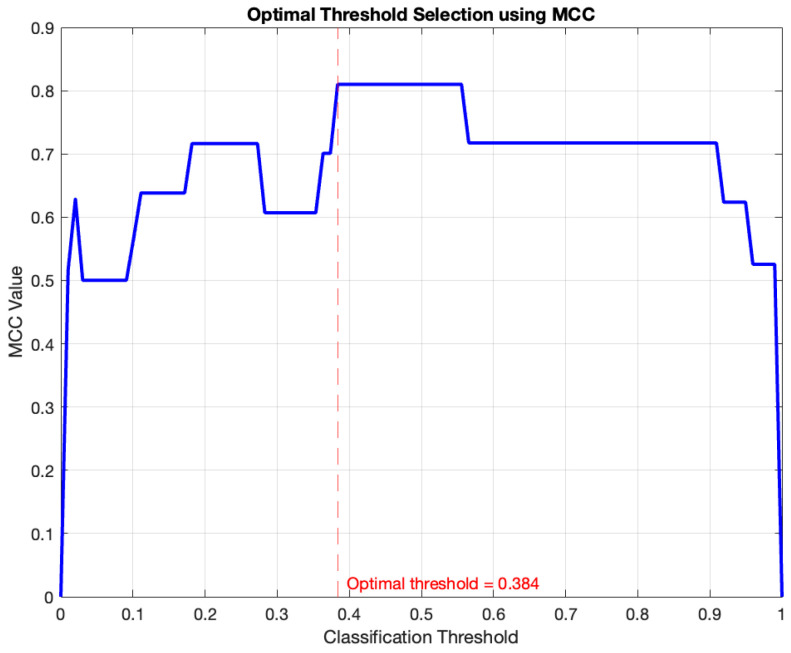
Determination of the optimal threshold based on the MCC method. The obtained cutoff point (0.384) corresponds to the red dashed line and is associated with an MCC of 0.81.

Figure 4 shows the graphical optimization process from which a threshold of 0.384 was obtained, associated with an MCC of 0.81.

**Figure 5:**
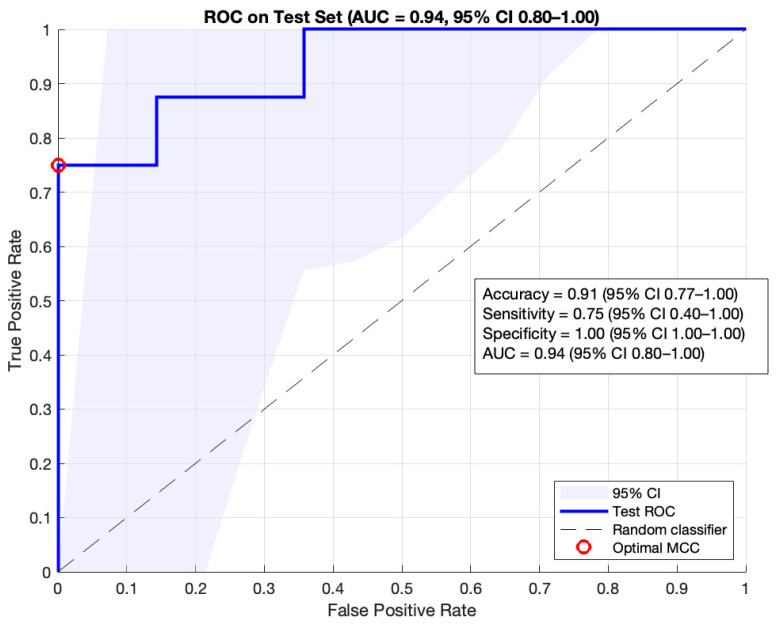
ROC Curve on the test set (blue curve). The optimal cutoff point (red circle) for the system’s performance, calculated using the MCC method, is associated with a sensitivity of 75% and a specificity of 100%.

Figure 5 shows the ROC curve of the classifier obtained for the test set, together with other model performance evaluation metrics. All results are presented with their corresponding 95% confidence interval (CI), calculated using the bootstrap method. The AUC obtained on the test set was 0.94 (95% CI: 0.80–1.00). At the selected cutoff point, a sensitivity of 75.00% (95% CI: 40–100%) and a specificity of 100.00% (95% CI: 100–100%) were achieved. Likewise, an accuracy of 91% (95% CI: 77–100%), an F1-score of 86% (95% CI: 57–100%) and a precision of 100% (95% C1: 100–100%) were obtained. Given the small size of the test set, the confidence intervals are relatively wide.

Complementing the assessment of test acceptability, a color map based on the gradient-weighted class activation mapping (Grad-CAM) method [37] is overlaid on the input image. This approach allows us to highlight the regions of the image that have the greatest influence on the prediction.

#### 2.3.3. Alert Generation and Decision Making

Once the patient’s data has been processed, the intelligent system assigns one of these labels to the performed spirometry test: “Acceptable Test” or “Unacceptable Test”.

Based on this information, the personnel responsible can make the pertinent decisions, such as the repetition of the test if necessary.

Likewise, the system makes the assigned label available to the clinical team responsible for the interpretation, which facilitates a more precise evaluation, supporting both the diagnosis and the patient’s follow-up.

## 3. Case Study

Next, a case study is proposed with the objective of exemplifying the system’s operation through a simple case that serves as proof of concept. To do this, data from a spirometry reserved in the test set were used, ensuring that these data was not employed during the system’s training. Specifically, an unacceptable test correctly classified by the system was randomly selected, allowing us to illustrate both the decision-making process and the usefulness of the Grad-CAM map in improving the model’s interpretability.

### 3.1. Data Collection

Table 5 shows the data for the spirometry test that has been selected for the proof of concept. It corresponds to a woman, who is 45 years old and has a BMI of 18.1 kg/m^2^.

**Figure 6:**
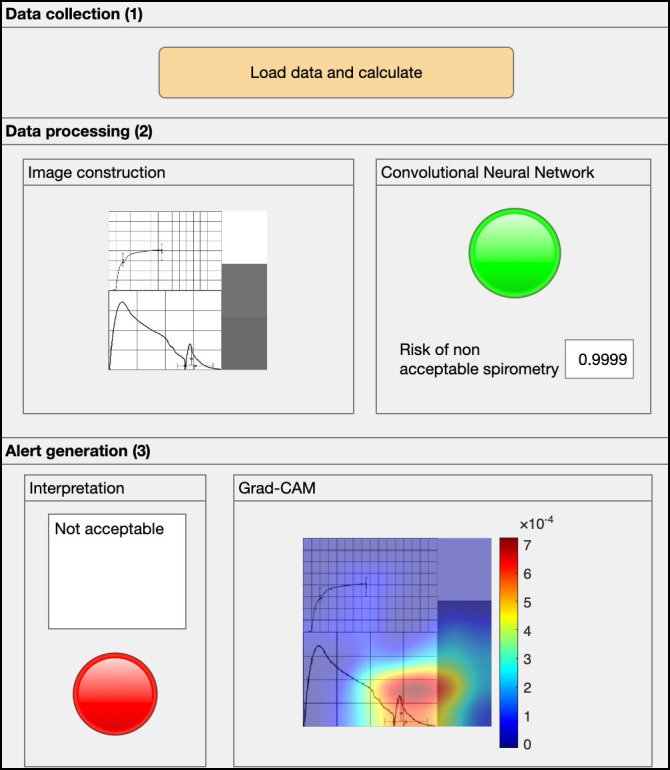
Screenshot of the user interface for the case study.

The spirometry report is introduced into the user interface through the Data collection panel (1), as shown in Figure 6.

With the objective of comparing the prediction made by the system, it must be indicated that the spirometry test performed by the patient was classified by the experts as unacceptable.

### 3.2. Data Processing

Once the data has been introduced into the application, the system processes it automatically. In the Data Processing panel (2) of the user interface shown in Figure 6, the generated image that serves as the input for the CNN is visualized. Subsequently, the output of the CNN provides the risk that the spirometry test is unacceptable, which is shown on the right side of the same panel, also illustrated in Figure 6. In this case, the obtained risk was ≈1.

### 3.3. Alert Generation and Decision Making

Based on this risk and considering the obtained threshold using the MCC method, the intelligent system classifies the test as unacceptable, as shown in the Alert Generation panel (3) on Figure 6. On the right side of the same panel, the Grad-CAM map is presented, superimposed on the input image, highlighting the zones with the greatest influence on the prediction. In this case, the highlighted area corresponds to a peak at the end of the flow-volume curve caused by coughing, thereby invalidating the test. This provides additional information about errors made during the test, allowing for guidance on corrective actions.

The system performs the classification correctly according to the test’s label. With this information, the responsible technician can take the pertinent actions, such as requesting the repetition of the test or indicating that it is unacceptable, thereby ensuring an adequate diagnosis and patient follow-up.

## 4. Discussion

Spirometry is the most widely used pulmonary function test for the diagnosis and follow-up of various respiratory diseases [1,2,3]. The increasing prevalence of chronic pathologies such as asthma and COPD has favored its implementation in primary care, with the aim of facilitating an early diagnosis [7,8,9]. Although it is a simple, safe, and non-invasive test, its correct execution requires the active cooperation of the patient and the supervision of trained personnel, thereby ensuring the validity of the results [1,10]. In this context, quality control is essential to prevent diagnostic and therapeutic errors. Currently, quality control largely relies on the visual inspection of the flow-volume and volume-time curves [1]; however, the subjective nature of this manual process, coupled with its dependence on specialized operator experience and time demands [12], presents a clear limitation.

Despite the growing availability of spirometers in primary care, the expansion of the test has not always been accompanied by the adequate training of the responsible personnel [2], which can compromise the quality and reliability of the results. Recently, AI has offered diverse decision-support tools in clinical practice, including intelligent systems based on CNNs for quality control in spirometry [12,23,24]. However, the number of studies is limited, and they present certain restrictions. Therefore, it is essential to promote the development of intelligent systems that help overcome the underutilization of this test in primary care.

First, there is high heterogeneity in the objectives of the classifiers designed. Walag et al. [23] not only classify the test as acceptable or not, but also evaluate specific parameters such as FEV_1_ and FVC, which can hinder overall interpretation. Bonthada et al. [24], on the other hand, focus on detecting specific errors, indicating the presence or absence of four types of errors: extra breaths, high extrapolated volume, early termination, or submaximal effort. Although this approach provides feedback on specific errors, it is insufficient as it does not cover other frequent failures that also invalidate the test, such as coughing during the first second, glottis closure, or effort variability. Consequently, these systems do not allow for comprehensive quality control, as they are unable to provide a complete classification of the test by themselves.

On the other hand, the published studies use different types of features as input to the intelligent systems. Walag et al. [23] use flow-time signals, Das et al. [12] rely on images of flow-volume curves, and Bonthada et al. [24] use images of flow-volume and volume-time curves. This heterogeneity limits the clinical applicability and integration of these systems into the regular workflow, as none directly leverage the spirometry reports, which constitute the standard information after the test is performed. Furthermore, these reports include relevant patient data, which could be incorporated to improve the system’s generalization, given that they influence the execution and validity of the spirometry test.

In this context, the present work proposes an innovative intelligent system based on a CNN to classify spirometry tests as acceptable or unacceptable. The system constructs an image from the spirometry reports obtained after the test, integrating flow-volume and volume-time curves along with patient demographic information. This approach allows for the development of a system that can be easily implemented in clinical practice without interrupting the usual workflow, providing effective support for quality control, especially in the visual inspection of curves, which is still considered the gold standard for assessing test acceptability. This feature sets our system apart from other existing quality control systems, which disregard visual inspection of the curves and focus instead on the validity of spirometric parameters, which are not always effective in determining the acceptability of the test. Furthermore, by incorporating the demographic information contained in the reports, the system provides a more complete and robust assessment of test quality.

The design of the graphical construct constitutes a central component of this work. For its construction, the spirometry report in PDF format is processed to extract the information necessary for classification. This includes the patient’s demographic data, in tabular format, and the corresponding spirometric curves. The first page of the PDF is converted into a PNG image, which is filtered to retain only the relevant information, removing the post-bronchodilator curve, and is then transformed into grayscale.

Subsequently, the sections corresponding to the flow-volume and volume-time curves are extracted from the image, generating two grayscale images that are combined into a single image. This image is then enriched with the patient’s demographic information, including sex, age, and BMI, with the aim of improving the classifier’s performance and leveraging all the relevant information contained in the report. The inclusion of these variables is based on both expert opinion and previous studies that demonstrate their influence on spirometry quality. For instance, Tan et al. [38] indicate that being female and young is associated with good quality maneuvers, while Lawrence et al. [39] observe that patients with a BMI ≥ 25 have a lower probability of performing unacceptable maneuvers. This strategy constitutes an innovation compared to previous studies, by efficiently integrating clinical and demographic information, creating a more robust classifier that considers the specific characteristics of each patient without interfering with the workflow of routine clinical practice.

One of the main challenges in developing intelligent systems for spirometry quality control is the availability of multicenter data from non-specialized settings. In this context, the studies by Das et al. [12] and Bonthada et al. [24] do not include data from spirometry tests performed in primary care. Conversely, Walag et al. [23] do use such data, though combined with information from other settings, which limits their specific applicability. In the present work, a database of 224 spirometry reports obtained during a training course in primary care centers is used. This selection ensures high-quality data for system training and allows for direct implementation in primary care, where the system could have a greater impact. Furthermore, the incorporation of relevant information contained in the reports, such as BMI, age, and sex, helps compensate for the limited amount of available data, thereby improving the classifier’s performance and generalization.

Also, a transfer learning strategy was adopted, using a CNN based on the ResNet-18 [32] model. This approach helps mitigate the problems associated with data scarcity, avoiding the random initialization of weights and achieving optimal results with the available information. ResNet-18 was selected for its high speed and accuracy, combined with a reduced number of parameters. This last feature reduces the risk of overfitting, which is commonly associated with more complex models, and facilitates the training of the network. However, the designed system is versatile and could be easily adapted to more complex networks, such as ResNet-50, depending on future needs.

During training, a strategy of dividing the database into training, validation, and test sets was applied. Unlike previous studies, such as that by Bonthada et al. [24], the evaluation of the system’s performance in this work was carried out using AUC, as well as the sensitivity and specificity calculated at the optimal cut-off point using the MCC method. This approach allows for a more complete assessment of the classifier’s performance, providing a high score, with 1 considered a perfect classification, if optimal results are achieved across all measures of the confusion matrix. The system achieved an AUC of 0.94 (95% CI: 0.80–1.00), with a sensitivity of 75% (95% CI: 40–100%) and a specificity of 100% (95% CI: 100–100%) at the optimal cut-off point, with an associated MCC of 0.81. These results surpass those reported in the literature; for instance, Das et al. [12] obtained an AUC of 0.93 for classifying curves as acceptable or unacceptable. Furthermore, the specificity obtained is higher than that reported by Walag et al. [23], Das et al. [12] and Bonthada et al. [24] who reported values between 85.0% and 96.0%.

In conclusion, the intelligent system developed in this work is presented as an effective tool to assist in the quality control of spirometry tests, especially in settings like primary care. The system allows maneuvers to be classified as acceptable or unacceptable, providing technicians with immediate feedback that facilitates the repetition of the test when necessary. This will make it possible to speed up the acquisition of at least three acceptable maneuvers, which are required to subsequently meet the repeatability criteria needed to ensure the diagnostic validity of the test. In this way, the tool not only optimizes efficiency in performing the test but also ensures compliance with the standards established by the ATS and the ERS [1,10,11], guaranteeing reliable spirometric interpretation.

Furthermore, the inclusion of an overlaid color map based on the Grad-CAM method improves the clinical interpretability of the designed system by highlighting areas that have the greatest influence on the classification. In the case of unacceptable tests, these maps help identify specific problems, making it easier to determine the type of error present. This helps to compensate for the lack of experience in interpreting spirometry tests, offering objective support to less-trained technicians. Furthermore, the accurate identification of test quality helps prevent diagnostic errors and reduces inter-operator variability.

Additionally, the system works directly with spirometry reports, processing and classifying them without requiring additional actions from the technicians, which ensures fluid integration into clinical workflows. In this way, it promotes greater efficiency and agility in diagnosis via spirometry, even in non-specialized settings, optimizing the care of patients with various respiratory diseases.

### Limitations

First, although the data used for training is of high quality, the database is relatively small, with only 224 records, which restricts its use with certain DL techniques and increases the risk of overfitting. To mitigate this limitation, a transfer learning strategy was applied, which allows for good results without requiring an extensive database. Nevertheless, the use of pre-trained networks reduces the model’s versatility and requires using a predetermined input image size, which can introduce distortions and affect the system’s performance. Furthermore, the database was constructed from previous studies and was not specifically designed for this work.

Although the system’s performance has been evaluated on an independent test set, it should be noted that its limited size (22 images) may compromise the robustness and generalizability of the results obtained. For this reason, future work will need to expand the database size and carry out an external validation process to ensure the proper functioning of the system before its incorporation into clinical practice.

Another relevant limitation is the heterogeneity of the spirometry equipment used in different clinical settings. This variability means that the spirometry reports differ in format depending on the equipment used. In this study, the reports come from spirometry tests performed with the Medikro^®^ Pro M915 equipment, which could make it difficult to generalize the system to settings where other devices are employed. Furthermore, the spirometry tests were conducted within the context of training courses, which can make it difficult for the system to be used by professionals with varying levels of experience. Therefore, in future work, it would be necessary to have a more diverse database, including reports obtained with different equipment and performed by operators with different skill levels, to make the system reliable for its use in diverse clinical settings.

Additionally, the spirometry reports contain a large amount of information that could be leveraged for the classification of tests. In this work, a graphical construct was designed that combines images of the spirometry curves with relevant patient demographic information (sex, age, and BMI), variables proven to influence spirometry quality. The study’s main contribution lies in this innovative design as input to the system; however, alternative representations could be explored to further optimize the classifier’s performance.

The system is a binary classifier that indicates whether the test is acceptable or unacceptable, without providing additional information such as the type of error made by the patient during the test. This may limit its use in clinical practice, as it does not indicate the reason for non-acceptability. In future research, the addition of a multiclass classifier will be explored to identify the errors made during testing, with the aim of guiding corrective actions.

Finally, it is important to highlight the lack of transparency of DL models based on CNNs, which can limit their acceptance in clinical practice. Although attempts have been made to mitigate this limitation using techniques like Grad-CAM, the interpretability of the results remains a challenge and constitutes the main barrier to the integration of these tools into clinical settings.

## 5. Conclusions

This work presents an intelligent system to support spirometry quality control, allowing tests to be classified as acceptable or unacceptable. Unlike previous approaches, the system operates directly on the complete spirometry reports, which facilitates its integration into daily clinical practice.

As an innovation, the system generates a graphical construct that integrates the spirometry curves, specifically the flow-volume curve and the volume-time curve, with relevant patient demographic information, such as sex, age, and BMI. This data integration maximizes the use of the information generated during the test, increasing the efficiency and robustness of the classifier, and mitigating problems associated with patient variability and limited availability of data for system training.

The system’s development was supported by a proprietary database collected in primary care and the use of transfer learning strategies via ResNet-18, as well as the use of advanced metrics like the MCC. This contributed to improving both the robustness and performance of the model. The results obtained are promising, with an AUC of 0.94 (95% CI: 0.80–1.00) and a specificity and sensitivity of 100.00% (95% CI: 100–100%) and 75.00% (95% CI: 40–100%), respectively, at the selected cut-off point.

Nevertheless, it is important to note that the system is still in the development phase and requires an exhaustive validation process before its implementation in clinical practice.

## Figures and Tables

**Table 1:** Label distribution.

Classification of the Test	Frequency	Percentage (%)
Unacceptable (0)	79	35.53
Acceptable (1)	145	64.47
Total	224	100

**Table 2:** Summary of demographic variables.

Variable	Type	Comment
Sex	Categorical	Man (0)/Woman (1)
Age (Years)	Numerical	-
BMI (kg/m^2^)	Numerical	-

**Table 3:** Data augmentation strategy.

Data Augmentation Strategy	Range	Comment
Rotation	[−10, 10]	Applies random rotations to the images within the specified range.
Horizontal translation	[−5, 5]	Shifts the image horizontally within the specified pixel range.
Vertical translation	[−5, 5]	Shifts the image vertically within the specified pixel range.
Horizontal scaling	[0.9, 1.1]	Scales the image in the horizontal direction by multiplying it by a random value within the specified range.
Vertical scaling	[0.9, 1.1]	Scales the image in the vertical direction by multiplying it by a random value within the specified range.

**Table 4:** Configuration for CNN training.

Hyperparameter	Value	Comment
Optimizer	Adam	-
Mini-batch size	64	-
Maximum epochs	100	-
Learning rate	0.0001	-
Validation patience	20	The maximum value of consecutive iterations that the validation error is allowed to not improve before the training interruption is activated.
Output network	Best validation error	It allows for the selection of the neural network parameters that correspond to the iteration which has the lowest error on the validation set.

**Table 5:** Patient data.

Variable	Value
Sex	Woman
Age (Years)	45
BMI (kg/m^2^)	18.1

## Data Availability

The dataset is not publicly available as the research is ongoing. The data will be made available from the authors upon reasonable request.
